# Hyponatremia is Associated with Fluid Imbalance and Adverse Renal Outcome in Chronic Kidney Disease Patients Treated with Diuretics

**DOI:** 10.1038/srep36817

**Published:** 2016-11-14

**Authors:** Lee Moay Lim, Ni-Chin Tsai, Ming-Yen Lin, Daw-Yang Hwang, Hugo You-Hsien Lin, Jia-Jung Lee, Shang-Jyh Hwang, Chi-Chih Hung, Hung-Chun Chen

**Affiliations:** 1 Division of Nephrology, Department of Internal Medicine, Kaohsiung Medical University Hospital, Kaohsiung Medical University, Kaohsiung, Taiwan; 2 Department of Obstetrics and Gynecology, Kaohsiung Chang Gung Memorial Hospital, Chang Gung University, College of Medicine, Taiwan; 3 Faculty of Renal Care, College of Medicine, Kaohsiung Medical University, Kaohsiung, Taiwan; 4 Department of Internal Medicine, Kaohsiung Municipal Ta-Tung Hospital, Kaohsiung, Taiwan

## Abstract

Chronic kidney disease (CKD) is frequently complicated with hyponatremia, probably because of fluid overload or diuretic usage. Hyponatremia in CKD population is associated with increased mortality, but the effect on renal outcome was unknown. We investigated whether hyponatremia is associated with fluid status and is a prognostic indicator for adverse outcomes in a CKD cohort of 4,766 patients with 1,009 diuretic users. We found that diuretic users had worse clinical outcomes compared with diuretic non-users. Hyponatremia (serum sodium <135 mEq/L) was associated with excessive volume and volume depletion, measured as total body water by bioimpedance analysis, in diuretic users, but not in diuretic non-users. Furthermore, in Cox survival analysis, hyponatremia was associated with an increased risk for renal replacement therapy (hazard ratio, 1.45; 95% CI, 1.13–1.85, *P* < 0.05) in diuretic users, but not in diuretic non-users (*P* for interaction <0.05); restricted cubic spline model also showed a similar result. Hyponatremia was not associated with all-cause mortality or cardiovascular event whereas hypernatremia (serum sodium >141 mEq/L) was associated with an increased risk for all-cause mortality. Thus, hyponatremia is an indicator of fluid imbalance and also a prognostic factor for renal replacement therapy in CKD patients treated with diuretics.

Chronic kidney disease (CKD) causes dysfunction in regulating water homeostasis because of a reduced glomerular filtration rate (GFR)^1^. Volume overload is highly prevalent in patients with CKD and a 10% to 30% increase in extracellular fluid can be detected, even in the absence of overt edema^2^. The disorder in homeostasis resulted in hypertension, electrolyte imbalance and edema. Volume overload has been associated with CKD progression and cardiovascular disease (CVD) related morbidity or mortality^3^. Diuretics are essential in treating fluid balance, blood pressure control, prevention of hyperkalemia and urine amount regulation in CKD population^4^,^5^,^6^. However, several studies have reported negative outcomes of diuretic usage on renal function, mortality, and hospitalization in patients with heart failure^7^,^8^,^9^,^10^,^11^. In acute kidney injury (AKI), loop diuretics exert no significant effect on renal recovery, the need for dialysis, or mortality^12^,^13^,^14^,^15^. In CKD, the long-term effect of diuretic usage in correction of volume overload has not been thoroughly studied^16^.

Sodium imbalance could be secondary to diuretic usage, particularly in patients with CKD, because the ability of kidneys to regulate dilution and concentration becomes impaired as renal disease progressing^2^. Hyponatremia could be a consequence of fluid overload or a consequence of diuretic usage in these patients. Various epidemiological studies have documented an association between hyponatremia and increased mortality from diseases involving fluid overload and diuretic usage, such as congestive heart failure (CHF) and liver cirrhosis^17^,^18^,^19^,^20^,^21^. Recently, Kovesdy *et al*. discovered that both lower and higher serum sodium levels are associated with higher mortality in patients with CKD, independent of CHF and liver disease^22^, but renal outcome and the degree of diuretic usage were not reported. Due to the unique disease characteristic that are susceptible to sodium imbalance, hyponatremia could be prognostic indicator in patients with CKD. Thus, the aim of our study was to determine whether diuretic usage and the related hyponatremia are associated with fluid imbalance and are predictive of adverse clinical outcomes, including renal outcomes, in patients with CKD.

## Results

### Baseline characteristics and clinical outcomes of the diuretic users and diuretic non-users

Table 1 showed the baseline characteristics of diuretic users and non-users which comprised of 1,009 and 3,757 respectively. The diuretic users showed higher percentage of CHF, Diabetes mellitus (DM), CVD and severe liver disease (SLD). They exhibited a significantly higher mean blood pressure, urine protein-to creatinine ratio (UPCR) and HbA1c level (*P* < 0.05). At the same time, lower estimated glomerular filtration rate (eGFR), serum hemoglobin and albumin were observed in diuretic users (Table 1). However, only male diuretic users showed higher total body water (TBW) (55.5 ± 8.2% vs 54.0 ± 5.7%). Diuretic users also showed higher percentage of renal replacement therapy (RRT) and CVD.

### Baseline characteristics of diuretic users according to serum sodium

Table 2 showed the baseline characteristics of diuretic users. The mean age was 64.0 ± 13.5 years and 49.6% were female. Diuretic users were divided according to mean serum sodium ± 1 SD into 4 groups: Na <135 mEq/L, Na 135–138 mEq/L, Na 138–141 mEq/L, and Na >141 mEq/L (Table 2). Diuretic users with Na <135 mEq/L were more likely than those with Na 138–141 mEq/L to experience CHF, DM, and CVD, have higher blood glucose levels, HbA1C and UPCR, and have lower albumin concentrations. Diuretic usage did not differ among the Na groups (Table 2). In male diuretic users, Na <135 mEq/L was associated with lowest TBW.

### Association between hyponatremia and total body water in diuretic users

Table 3 showed the variables associated with hyponatremia (Na<135 mEq/L) in diuretic users. To understand whether hyponatremia could be an indicator of fluid status, we measured TBW by using the bioimpedance method in 498 randomly selected diuretic users and performed logistic regression to determine the nonlinear association between serum sodium and TBW. Our results showed that the lowest and highest quartiles of TBW were associated with hyponatremia (Table 3). Age, CVD, HbA1c and BMI also showed positive correlation with hyponatremia but not thiazide diuretics. No association between serum sodium and TBW was noted in diuretic non-users (Supplementary Table 1). CVD, eGFR, albumin, HbA1c and BMI showed positive correlation with hyponatremia in diuretic non-users.

### Association between serum sodium and clinical outcomes in diuretic users

**Figure 1:**
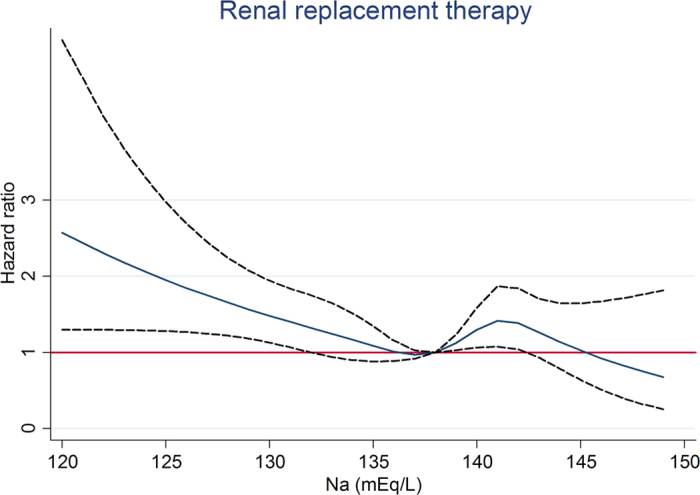
Association between serum sodium and renal replacement therapy by restricted cubic spline model in diuretic users.

**Figure 2:**
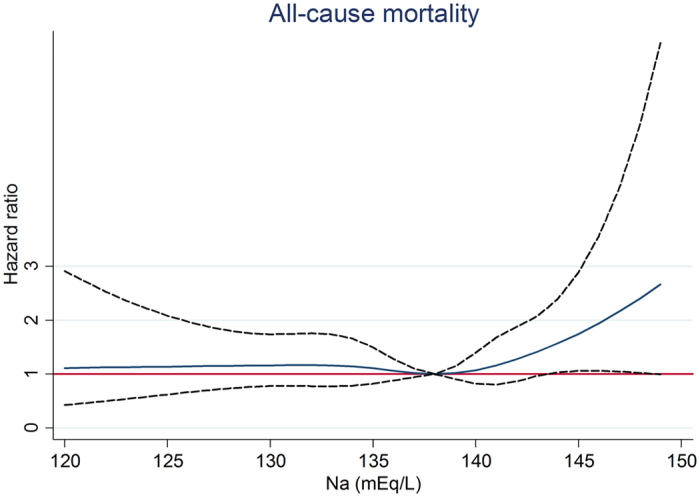
Association between serum sodium and all-cause mortality by restricted cubic spline model in diuretic users.

In diuretic users, after a median follow-up of 1070 days, the RRT event was higher in Na <135 mEq/L, which was 113 (62.4%) (Table 4). In fully-adjusted competing risk Cox regression model, compared with Na 135–138 mEq/L, Na <135 mEq/L was associated with an adjusted HR of 1.45 (95% CI, 1.13–1.85; *P* < 0.05) for RRT, but not associated with all-cause mortality and cardiovascular events (Table 4). Na >141 mEq/L was associated with a trend of increased risk for RRT. When compared with Na 138–141 mEq/L, Na >141 mEq/L had an adjusted HR of 1.59 (95% CI, 1.09–2.32; *P* < 0.05) for all-cause mortality. In analysis of serum sodium and outcomes as restricted cubic splines, we observed that Na <132 mEq/L was associated with a higher risk for RRT (Fig. 1) whereas Na >143 mEq/L was associated with a higher risk for all-cause mortality (Fig. 2).

### Association between serum sodium and clinical outcomes in diuretic non-users and interaction between diuretic use and serum sodium

We studied the prognostic effect of sodium in diuretic non-user (Supplement Table 2). The percentage of Na <135 mEq/L in diuretic non-users was 17.3% similar to that in diuretic users, 17.9%. Na <135 mEq/L was associated with increased risks of RRT, all- cause mortality and cardiovascular events in unadjusted models; however, the result became non-significant after adjustment (Supplement Table 2). In analysis of serum sodium and outcomes as restricted cubic splines, we also did not observe a significant association (Supplement Figures 1, 2 and 3). The interaction between diuretic use and serum sodium for RRT was significant with a p value of 0.017 and 0.038, when serum sodium treated as a categorical variable or a continuous variable, respectively.

### Sensitivity test for the association between serum sodium and clinical outcomes

We tested other grouping methods as stated in the method. In diuretic users, compared with Na 135–141 mEq/L, Na <135 mEq/L was associated with an increased risk of RRT and Na >141 mEq/L was associated with an increased risk of all-cause mortality (Supplement Table 3). In analysis by restricted cubic spline model with different knots, we also observed that Na <132 mEq/L was associated with a higher risk for RRT (Supplement Figure 4) whereas Na >143 mEq/L was associated with a higher risk for all- cause mortality (Supplement Figure 5). In diuretic non-users, serum sodium treated as a categorical variable (3 groups) or as a continuous variable with different knots showed similar results (data not shown). The interaction between diuretic use and serum sodium for RRT was also significant with a p value of 0.024 and 0.036, when serum sodium treated as a categorical variable or a continuous variable, respectively (data not shown).

## Discussion

We investigated the diuretic usage, clinical outcomes, and prognostic effect of serum sodium among patients in a CKD cohort. We found that hyponatremia is associated with imbalance of TBW in diuretic users. In investigating the prognostic effect of diuretic- related hyponatremia, we revealed, for the first time, that serum sodium <135 mEq/L is independently associated with a higher risk for RRT in diuretic users, but not in diuretic non-users. Moreover, we also observed that serum sodium >141 mEq/L is associated with an increased risk for all-cause mortality.

Previous studies on the effects and outcomes of diuretic usage have mainly focus on patients with AKI or edematous diseases other than kidney disease. Using diuretics to treat AKI provides no clear benefits in the recovering of kidney function or preventing mortality^23^ other than maintaining urine output^13^. Moreover, diuretic usage by critically ill patients with AKI was associated with an increased risk of death and non-recovery in renal function^24^. Observational studies have shown that diuretic usage by patients with heart failure is associated with reduced renal function, the progression of heart failure and increased mortality^25^,^26^,^27^. Compared with paracentesis, diuretic usage was associated with a higher incidence of renal impairment and other complications in patients with liver cirrhosis and ascites^28^. These data suggest that diuretic usage could be harmful to patients with extreme volume changes.

Clinical trials in patients with hypertension have yielded controversial results regarding the effects of diuretic usage on clinical outcomes. The Antihypertensive and Lipid-Lowering Treatment to Prevent Heart Attack Trial (ALLHAT) reported that thiazide diuretics were not inferior to calcium channel blockers and angiotensin- converting enzyme inhibitors in preventing all-cause mortality and were superior in preventing CVD^29^. The renal outcomes of these drugs did not differ^30^. However, in the Avoiding Cardiovascular Events in Combination Therapy in Patients Living with Systolic Hypertension (ACCOMPLISH) study, benazepril plus hydrochlorothiazide, compared with benazepril plus amlodipine, were more strongly associated with hypotension and higher risks of the progression of CKD, cardiovascular morbidity, and mortality^31^. These data suggest that thiazide diuretics could be harmful if they cause volume depletion.

Little research has examined the association between diuretic usage and outcomes in CKD comparatively. Diuretics have considerable therapeutic importance in treatment volume overload of CKD patients, but may possess direct nephrotoxicity, particularly tubulointerstitial injury^32^ and impaired renal vaso-relaxation^33^. The overuse of diuretics causes volume depletion and subsequently increased sympathetic activity^34^ and stimulates the renin-angiotensin-aldosterone system^34^, as evidenced by animal studies. Both local renal effects and systemic neuro-hormonal effects impair renal function. In patients with hypertension and GFR <60 mL/min/1.73 m^2^, thiazide diuretics were not inferior to 2 other drugs in reducing end-stage renal disease in the ALLHAT study^35^, and benazepril plus hydrochlorothiazide were not associated with hypotension or a higher risk of the progression of CKD in the ACCOMPLISH study^31^. No previous studies investigated the impact of loop diuretics in patients with CKD. In our study, we discovered that diuretic users (80% loop diuretics) had worse clinical outcomes. However, the causal relationship between them could not be determined.

In patients with CKD and volume overload, diuretic usage may be a necessary evil in managing volume status. High prevalence of volume overload in CKD patients lead to unavoidable diuretic usage. Volume status could be measured by using the bioimpedance method^36^, which reflects the status of hydration quantitatively. However, this is not routinely use in daily practice. We found hyponatremia under diuretic usage could become a clinical parameter which indicates the increased TBW (increased fluid status despite diuretic use) or decreased TBW (likely because of too much diuretic use). Hyponatremia with increased TBW denotes water and salt retention because of impaired renal excretion and activation of the neuro-hormonal mechanisms^37^. The increased fluid status would increase renal efferent pressure and decrease renal blood^38^. Our previous study had shown that fluid overload is associated with worse renal outcome^36^. Conversely, hyponatremia with decreased TBW suggests volume depletion under diuretic usage,

which enhances arginine-vasopressin (AVP) and renin-angiotensin secretion^39^. Acute volume depletion is well-known as a cause of acute renal injury^40^ and chronic volume depletion could leads to chronic tubulointerstitial injury and CKD^41^. Volume depletion also decreases renal perfusion and increases the susceptibility to analgesics and nephrotoxic agents. Thus, hyponatremia indicates imbalanced volume status and could be associated with renal function progression.

Hyponatremia could also indicate an impaired renal dilution capability in CKD patients. As CKD progresses, renal sodium loss because of impaired tubular reabsorption and osmotic disequilibrium between the luminal fluid and medullary interstitial impair dilution^42^. CKD is also associated with increased AVP secretion and experimental evidence has demonstrated that AVP is critical in initiating and exacerbating renal damage^43^. A sustained stimulation of vasopressin receptors induced intrarenal renin- angiotensin system activation, glomerular hyperfiltration, and hypertrophy, causing proteinuria and glomerulosclerosis^43^. However, our data demonstrated that hyponatremia in diuretic non-users was associated with lower eGFR but was not associated adverse clinical outcomes. Besides, thiazide diuretics, which inhibit sodium transport in the distal tubule, could prevent the maximal dilution of urine^44^. But we found no association between hyponatremia and thiazide diuretics in diuretic users. These data suggest that impaired renal dilution capability could not explain the prognostic effect of hyponatremia in diuretic users.

Hyponatremia may also reflect concurrent heart failure, which may impose additive deleterious effects on poor renal outcomes. Fluid overload as indicated by hyponatremia could cause cardiomyocyte elongation and dysfunction during the left ventricle remodeling^45^. Our data demonstrated that hyponatremia was associated with cardiovascular disease in patients with CKD. Hoorn *et al*. demonstrated that hyponatremia predicts declining creatinine clearance in patients with severe heart failure^46^. The concourse of hyponatremia and renal dysfunction has been shown to be associated with heart and liver failure^47^. Thus, cardiorenal syndrome can cause a vicious cycle in which deteriorating heart function accelerates the reduction in kidney function via the neurohormonal pathways^48^.

Hyponatremia is an independent prognostic factor for morbidity and mortality in heart failure^19^,^49^ and maintenance hemodialysis^17^,^50^. Kovesdy *et al*. concluded that lower serum sodium (<135.9 mEq/L) and higher serum sodium (>145 mEq/L) were associated with higher mortality in a large cohort of patients with non–dialysis-dependent CKD^22^. In our CKD cohort, the lack of association between hyponatremia and mortality could be attributed to the fact that renal replacement therapy could resolve the fluid overload eventually in patients with advanced CKD^51^. Our data also reconfirmed the association between hypernatremia and mortality in CKD patients. Hypernatremia (serum sodium >145 mEq/L) was much less than hyponatremia (serum sodium <135 mEq/L), 3% vs 17%. Patients with hypernatremia could be associated with acute complications and death, rather than RRT.

Our study had several limitations. First, as an observational cohort, our ability to elucidate definite causal links was limited. Second, total body water measured by bioelectrical impedance analysis was not equal to extracellular fluid, though we had found a good correlation. Third, CV events were recorded in only one hospital, which might have caused an underestimation. Fourth, variations in serum sodium levels caused by daily intake, nutritional status, and medication use during the follow-up period could have confounded the results. However, our purpose was to apply serum sodium as a prognostic factor, not a causal factor. Fifth, the smaller sample size of the 2 extreme serum sodium levels and relatively short follow-up duration could have resulted in lower statistical power, which might account for the weak association with mortality and CV events and the inability to differentiate the effects of loop and thiazide diuretics.

In conclusion, diuretic users have adverse clinical outcomes in CKD population. Hyponatremia is associated with imbalanced TBW and is a prognostic indicator for RRT in diuretic users, but not in diuretic non-users. Hyponatremia was not associated with all- cause mortality or cardiovascular event whereas hypernatremia was associated with an increased risk for all-cause mortality. Therefore, the serum sodium levels of patients with CKD who are being treated with diuretics should be routinely evaluated. Additionally, interventions other than diuretics aimed at achieving optimal fluid status should be considered. Whether the mechanism behind hyponatremia is diuretic-induced direct renal injury or neuro-hormonal activation requires further study.

## Methods

### Participants and Measurement

Between November 11, 2002, and June 30, 2009, 5,047 patients who were screened through an integrated CKD care program at 2 affiliated hospitals of Kaohsiung Medical University in Southern Taiwan were included in the CKD cohort and followed until July 31, 2010. Integrated CKD care program Kaohsiung for delaying dialysis (ICKD) study was designed as a prospective cohort study to investigate the impact of an integrated CKD care program on clinical outcomes in patients with CKD stages 1–5 not on dialysis. CKD was staged according to K/DOQI definitions and the estimated glomerular filtration rate (eGFR) was calculated using the equation of the 4-variable Modification of Diet in Renal Disease (MDRD) Study. A total of 123 patients who were lost to follow-up within 3 months and 158 patients with incomplete medication information were excluded. Of the patients included, 3,659 were treated as part of the integrated care program and 1,107 received regular care. A total of 4,766 patients with CKD between stages 1 and 5 were eligible for this study. The study protocol was approved by the Institutional Review Board of Kaohsiung Medical University Hospital (KMUH-IRB-990198). Written informed consent was obtained from patients and all clinical investigations were conducted according to the principles expressed in the Declaration of Helsinki.

Baseline variables included demographic features, medical history, examination findings, laboratory data, and medication history. The demographic features were the baseline records when patients enrolled in the CKD care program. The medical history was obtained by reviewing doctor charts. DM and hypertension were defined according to clinical diagnoses and prescribed medications. CVD was defined as a clinical diagnosis of heart failure, acute or chronic ischemic heart disease, or cerebrovascular disease. Diuretic use was defined as loop diuretics exceeding equivalent furosemide 39 mg per day or thiazide diuretics exceeding equivalent trichlormethiazide 3.9 mg per day for more than half of the observation period. Other medication use was also defined as treatment for more than half of the observation period. To prevent variability, electrolytes including serum sodium were collected 3 months before and after enrollment and were averaged. To study the association of clinical outcomes with 2 ends of sodium level, patients were divided into 4 groups according to serum sodium levels, with cut-off values of 135 mEq/L, 138 mEq/L, and 141 mEq/L equal to mean −1 standard deviation (SD), mean, and mean +1 SD, respectively. We also examined serum sodium as a continuous predictor using restricted cubic spline analysis^52^.

Bioelectrical impedance analysis measures the change in impedance of electrical signals, which travel more rapidly through water and lean body mass than through fat body mass. The device used in this study was the InBody 230 (Biospace Co Ltd, Korea), which uses 2 frequencies (20 k and 100 kHz). Our preliminary data demonstrated that the first and fourth quartiles of TBW (expressed as % of body weight) were associated with adverse clinical outcomes. Accordingly, we defined decreased TBW as the first quartile (<25%) and increased TBW as the fourth quartile (>75%).

### Outcomes

Three outcomes were assessed: all-cause mortality, renal replacement therapy (RRT), and cardiovascular events. Survival status and cause of death were ascertained in a death certificate review by using charts and the National Death Index. Cardiovascular events were defined as the development of acute coronary syndrome or acute stroke, hospitalization for peripheral arterial occlusion disease or congestive heart failure, and death by these causes. The development of cardiovascular events was ascertained by reviewing charts. RRT was defined as the initiation of hemodialysis, peritoneal dialysis, or renal transplantation and was ascertained by reviewing charts and catastrophic illness certificate.

### Statistical Analysis

**Figure 3:**
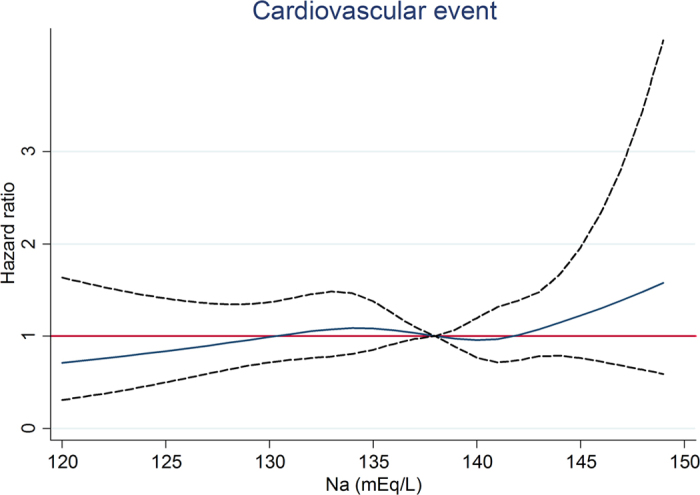
Association between serum sodium and cardiovascular event by restricted cubic spline model in diuretic users.

The baseline characteristics of all patients are expressed as percentages for categorical data, in mean ± SD for continuous variables with approximately normal distribution, and median and interquartile ranges for continuous variables with skewed distribution. The association between hyponatremia (Na<135 mEq/L) and clinical variables including TBW in diuretic users was evaluated using logistic regression analysis. Competing risk Cox proportional hazard analysis was used to assess the relationship between serum sodium and clinical outcomes. The covariates were selected according to previous studies and our past publications, and the continuous variables with skewed distributions were log-transformed to obtain normal distributions. The adjusted covariates included age, gender, estimated glomerular filtration rate (eGFR), DM, cardiovascular disease, mean blood pressure, glycated hemoglobin, hemoglobin, albumin, cholesterol, urine protein-to-creatinine ratio, C-reactive protein, body mass index, angiotensin-converting enzyme inhibitor/angiotensin II receptor blocker, anti- hypertensive agents, oral anti-diabetic drug, statins, integrated CKD care and causes of renal diseases^53^. Serum sodium was treated as a categorical variable with the cuf-off at 135, 138, and 141 mEq/L (the 16.6^th^, 50^th^, and 83.3^th^ percentile) in Table 4 and as a categorical variable with the cuf-off at 135 and 141 mEq/L (the 16.6^th^ and 83.3^th^ percentile) in Supplement Table 3. Serum sodium was treated as a continuous variable with 5 knots at 130, 135, 138, 141, and 143 (the 5^th^, 16.6^th^, 50^th^, and 83.3^th^, and 95^th^ percentile) in Figs 1, 2 and 3 and Supplement Figures 1, 2 and 3 and as a continuous variable with 5 default knots at 130, 136, 138, 140, and 143 (the 5^th^, 27.5^th^, 50^th^, 72.5^th^, and 95^th^ percentile) in Supplement Figures 4–6. *P* < 0.05 was considered statistically significant. Statistical analysis was performed with STATA 12.0 software (Stata Corp LP, College Station, TX, USA) and the Statistical Package for Social Sciences, Version 21.0 for Windows (SPSS Inc., Chicago, IL, USA).

## Additional Information

**How to cite this article**: Lim, L. M. *et al*. Hyponatremia is Associated with Fluid Imbalance and Adverse Renal Outcome in Chronic Kidney Disease Patients Treated with Diuretics. *Sci. Rep*. **6**, 36817; doi: 10.1038/srep36817 (2016).

**Publisher’s note:** Springer Nature remains neutral with regard to jurisdictional claims in published maps and institutional affiliations.

## Supplementary Material

Supplementary Information

## Figures and Tables

**Table 1:** Baseline characteristics and clinical outcomes of the diuretic users and diuretic non-users.

	Diuretic non-users (n = 3757)	Diuretic users (n = 1009)	*p*
**Demographics data**			
Age, yrs	61.2 ± 14.8	64.0 ± 13.5	<0.001
Female, n (%)	1573 (41.9%)	500 (49.6%)	<0.001
Congestive heart failure, n (%)	303 (8.1%)	279 (27.7%)	<0.001
Diabetes mellitus, n (%)	1297 (34.5%)	661 (65.5%)	<0.001
Cardiovascular disease, n (%)	774 (20.6%)	425 (42.1%)	<0.001
Severe liver disease, n (%)	167 (4.4%)	66 (6.5%)	0.006
Body mass index, kg/m^2^	24.5 ± 4.0	24.9 ± 3.8	0.002
Total body water (male), %	54.0 ± 5.7	55.5 ± 8.2	0.036
Total body water (female), %	49.0 ± 6.3	49.6 ± 6.5	0.374
Mean blood pressure, mmHg	99.2 ± 13.9	100.4 ± 14.8	0.030
Integrated CKD care	2840 (75.6%)	731 (72.4%)	0.116
**Causes of renal disease**			<0.001
Glomerulonephritis	1617 (43%)	249 (24.7%)	
Tubular interstitial disease	361 (9.6%)	36 (3.6%)	
Diabetes mellitus	1115 (29.7%)	584 (57.9%)	
Hypertension	399 (10.6%)	87 (8.6%)	
Other	265 (7.1%)	53 (5.3%)	
**Laboratory values**			
eGFR, ml/min/1.73 m^2^	33.7 ± 25.7	25.1 ± 22.3	<0.001
UPCR, mg/g	1026 (351–2106)	2089 (941–3715)	<0.001
Hemoglobin, g/dL	11.4 ± 2.5	10.2 ± 2.2	<0.001
Albumin, g/dL	3.9 ± 0.6	3.5 ± 0.6	<0.001
CRP, mg/dL	1.4 (0.4–6.5)	1.6 (0.5–6.2)	0.179
HbA1c, %	6.4 ± 1.6	7.0 ± 1.9	<0.001
**Outcomes**			
Renal replacement therapy, n (%)	949 (25.3%)	520 (51.5%)	<0.001
All-cause mortality, n (%)	561 (14.9%)	255 (25.3%)	<0.001
Cardiovascular events, n (%)	415 (11.0%)	273 (27.1%)	<0.001

Abbreviations: eGFR, estimated glomerular filtration rate; CRP, C-reactive protein; UPCR, Urine protein-to- creatinine ratio. Continuous variables are expressed as mean ± standard deviation or median (inter-quartile range), and categorical variables are expressed as percentage. p < 0.05 indicates a significant difference between diuretic non-user and diuretic user.

**Table 2:** Baseline characteristics of diuretic users according to serum sodium.

	All (n = 1009)	Na (mEq/L)				*p* for trend
		<135 (n = 181)	135–138 (n = 332)	138–141 (n = 335)	>141 (n = 161)	
**Demographics and Medical History**						
Age, year	64.0 ± 13.5	65.1 ± 11.2	63.4 ± 13.9	62.6 ± 14.6	66.8 ± 12.0	0.360
Female, n (%)	500 (49.6%)	97 (53.6%)	165 (49.7%)	151 (45.1%)	87 (54.0%)	0.607
CHF, n (%)	279 (27.7%)	68 (37.6%)	91 (27.4%)	75 (22.4%)	45 (28.0%)	0.011
DM, n (%)	661 (65.5%)	138 (76.2%)	220 (66.3%)	204 (60.9%)	99 (61.5%)	0.001
CVD, n (%)	197 (19.5%)	97 (53.6%)	148 (44.6%)	119 (35.5%)	61 (37.9%)	<0.001
SLD, n (%)	66 (6.5%)	10 (5.5%)	26 (7.8%)	22 (6.6%)	8 (5.0%)	0.679
BMI, kg/m^2^	24.9 ± 3.8	24.2 ± 3.6	24.9 ± 3.9	25.3 ± 3.8	25.1 ± 3.9	0.010
Total body water (male), %	55.5 ± 8.2	54.4 ± 9.1	55.2 ± 7.7	55.9 ± 8.1	57.2 ± 8.2	0.216
Total body water (female), %	49.6 ± 6.5	49.7 ± 5.5	49.4 ± 6.8	50.1 ± 7.5	48.2 ± 4.7	0.611
Mean BP, mmHg	100.4 ± 14.8	99.9 ± 14.3	100.4 ± 14.9	101.3 ± 14.9	98.8 ± 14.8	0.612
Integrated CKD care	708 (70.2%)	123 (68.0%)	238 (71.7%)	235 (70.1%)	112 (69.6%)	0.278
**Primary renal disease**						0.015
Glomerulonephritis	249 (24.7%)	37 (20.4%)	73 (22.0%)	107 (31.9%)	43 (26.7%)	
Tubular interstitial disease	36 (3.6%)	7 (3.9%)	8 (2.4%)	11 (3.3%)	10 (6.2%)	
Diabetes mellitus	584 (57.9%)	115 (63.5%)	194 (58.4%)	170 (50.7%)	94 (58.4%)	
Hypertension	87 (8.6%)	13 (7.2%)	34 (10.2%)	32 (9.6%)	8 (5.0)	
Other	53 (5.3%)	9 (5.0%)	23 (6.9%)	15 (4.5%)	6 (3.7%)	
**Laboratory values**						
eGFR, ml/min/1.73 m^2^	25.1 ± 22.3	23.2 ± 20.5	23.6 ± 20.9	28.5 ± 24.9	23.1 ± 20.7	0.537
UPCR, mg/g	2089(941–3715)	2477(1238–3715)	2106(899–4399)	1870(752–3690)	1869(973–3709)	0.043
Hemoglobin, g/dL	10.2 ± 2.2	9.9 ± 2.1	10.3 ± 2.1	10.5 ± 2.3	10.0 ± 2.1	0.595
Albumin, g/dL	3.5 ± 0.6	3.4 ± 0.6	3.5 ± 0.6	3.6 ± 0.6	3.6 ± 0.6	0.003
CRP, mg/dL	1.6 (0.5–6.2)	1.74 (0.44–6.59)	1.74 (0.5–7.79)	1.44 (0.50–4.68)	1.81 (0.46–6.10)	0.468
Sodium, mEq/L	137.5 ± 3.9	131.4 ± 3.1	136.5 ± 1.1	139.4 ± 0.9	142.5 ± 1.6	<0.001
Potassium, mEq/L	4.3 ± 0.6	4.3 ± 0.6	4.3 ± 0.6	4.3 ± 0.7	4.3 ± 0.7	0.362
HCO3, mg/dL	21.3 ± 4.6	20.5 ± 4.4	21.2 ± 4.5	21.7 ± 4.4	21.4 ± 4.9	0.786
Phosphorus, mg/dL	4.7 ± 1.3	4.7 ± 1.4	4.8 ± 1.3	4.5 ± 1.2	4.7 ± 1.2	0.476
Calcium, mg/dL	8.8 ± 0.8	8.7 ± 0.8	8.8 ± 0.7	8.8 ± 0.8	9.0 ± 0.7	0.268
Cholesterol, mg/dL	203.9 ± 67.8	202.4 ± 72.0	203.4 ± 72.5	204.9 ± 63.5	204.6 ± 62.3	0.729
Blood glucose, mg/dL	124.5 ± 54.4	141.8 ± 68.0	123.5 ± 48.3	118.9 ± 52.4	118.5 ± 49.6	<0.001
HbA1c, %	7.0 ± 1.9	7.7 ± 2.4	7.0 ± 1.7	6.7 ± 1.8	6.9 ± 1.6	<0.001
**Medications, n (%)**						
Furosemide	867 (85.9%)	152 (84.0%)	294 (88.6%)	283 (84.5%)	138 (85.7%)	0.850
Thiazide	175 (17.3%)	36 (19.9%)	52 (15.7%)	59 (17.6%)	28 (17.4%)	0.760
ACEI/ARB	655 (64.9%)	118 (65.2%)	209 (63.0%)	235 (70.1%)	93 (57.8%)	0.680
Anti-HTN agents	801 (79.4%)	145 (80.1%)	274 (82.5%)	256 (76.4%)	126 (78.3%)	0.230
OAD agents	448 (44.4%)	103 (56.9%)	149 (44.9%)	124 (37.0%)	72 (44.7%)	<0.001
Statins	403 (39.9%)	74 (40.9%)	131 (39.5%)	142 (42.4%)	56 (34.8%)	0.312

Abbreviations: CHF, congestive heart failure; DM, Diabetes mellitus; CVD, cardiovascular disease; SLD, Severe liver disease; BMI, Body mass index; BP, blood pressure; eGFR, estimated glomerular filtration rate; CRP, C-reactive protein; UPCR, Urine protein-to-creatinine ratio; ACEI, angiotensin-converting enzyme inhibitor; ARB, angiotensin II receptor blocker; Anti-HTN, anti-hypertensive; OAD, Oral antidiabetic drug. Continuous variables are expressed as mean ± standard deviation or median (inter-quartile range), and categorical variables are expressed as number and percentage.

P for trend < 0.05 indicates a significant trend for increasing Na levels.

**Table 3:** Logistic regression for sodium <135 mEq/L in diuretic users.

Variables	OR	95% CI	P
Age, year	1.032	1.005 to 1.060	0.022
Male	1.094	0.771 to 1.552	0.614
DM	1.207	0.778 to 1.871	0.401
CVD	1.614	1.137 to 2.290	0.007
Total body water			
1^st^ quartile	3.707	1.054 to 13.046	0.041
2^nd^ quartile	1.000	1.0	
3^rd^ quartile	1.048	0.273 to 4.019	0.946
4^th^ quartile	3.589	1.049 to 12.280	0.042
eGFR, ml/min/1.73 m^2^	0.999	0.989 to 1.009	0.835
Hb, g/dL	0.930	0.836 to 1.034	0.179
Albumin, mg/dL	0.863	0.616 to 1.210	0.393
CRPlog	1.030	0.860 to 1.233	0.748
HbA1c, %	1.241	1.130 to 1.363	0.005
UPCR log	1.165	0.790 to 1.719	0.442
Mean BP, mmHg	1.017	0.998 to 1.037	0.083
BMI, kg/m^2^	0.954	0.911 to 0.999	0.047
Thiazide (vs furosemide)	0.889	0.654 to 1.173	0.289

Abbreviations: as Table 1. *DM, Diabetes mellitus; CVD, cardiovascular disease; BMI, Body mass index; eGFR, estimated glomerular filtration rate; CRP, C-reactive protein; UPCR, Urine protein-to-creatinine ratio; Adjusted for age, gender, eGFR, diabetes mellitus, cardiovascular disease, mean blood pressure, HbA1c, hemoglobin, albumin, cholesterol, log-transformed urine protein to creatinine ratio, log-transformed C-reactive protein, body mass index, ACEI/ARB, anti-HTN agents, OAD agents and statins*.

**Table 4:** Associations between serum sodium and outcomes in diuretic users.

	Na (mEq/L)			
	<135	135–138	138–141	>141
**Renal replacement Therapy**				
Event	113 (62.4%)	174 (52.4%)	148 (44.2%)	85 (52.8%)
Unadjusted HR	1.33 (1.05–1.68)*	1 (reference)	0.80 (0.63–0.98)*	1.02 (0.79–1.32)
Adjusted HR	1.45 (1.13–1.85)*	1 (reference)	1.18 (0.94–1.48)	1.22 (0.83–1.71)
**All-cause mortality**				
Event	56 (30.9%)	86 (25.9%)	60 (17.9%)	53 (32.9%)
Unadjusted HR	1.21 (0.86–1.69)	1 (reference)	0.72 (0.52–1.00)	1.25 (0.89–1.77)
Adjusted HR	1.11 (0.79–1.58)	1 (reference)	0.87 (0.62–1.22)	1.38 (0.98–1.96)
**Cardiovascular event**				
Event	58 (32.0%)	89 (26.8%)	76 (22.7%)	50 (31.1%)
Unadjusted HR	1.22 (0.93–1.59)	1 (reference)	0.72 (0.56–0.94)*	1.04 (0.78–1.39)
Adjusted HR	1.06 (0.80–1.40)	1 (reference)	0.92 (0.70–1.19)	1.15 (0.86–1.54)

Adjusted for age, gender, eGFR, diabetes mellitus, cardiovascular disease, mean blood pressure, HbA1c, hemoglobin, albumin, cholesterol, log-transformed urine protein to creatinine ratio, log-transformed C-reactive protein, body mass index, ACEI/ARB, anti-HTN agents, OAD agents, statins, integrated CKD care and causes of renal diseases. *(*p* < 0.05) indicates a significantly different from reference group.
