# Supplementary material for: Hyponatremia is Associated with Fluid Imbalance and Adverse Renal Outcome in Chronic Kidney Disease Patients Treated with Diuretics
Source: Sci Rep. 2016 Nov 14;6:36817. doi: 10.1038/srep36817 (PMC5108044; doi:10.1038/srep36817)

Hyponatremia Is Associated with Fluid Imbalance and Adverse Renal Outcome in Chronic Kidney Disease Patients Treated with Diuretics

Lee Moay Lim, MD,[1+] Ni-Chin Tsai, MD,[1,2+] Ming-Yen Lin, PhD[1,4], Daw-Yang Hwang, MD,[1] Hugo You-Hsien Lin, MD,[1] Jia-Jung Lee, MD,[1] Shang-Jyh Hwang, MD,[1,4] Chi-Chih Hung, MD,[1*] Hung-Chun Chen, MD,[1,4]

[1]Division of Nephrology, Department of Internal Medicine, Kaohsiung Medical University Hospital, Kaohsiung Medical University, Kaohsiung, Taiwan

[2]Department of Obstetrics and Gynecology, Kaohsiung Chang Gung Memorial Hospital, Chang Gung University College of Medicine, Taiwan

[3]Department of Internal Medicine, Kaohsiung Municipal Hsiao-Kang Hospital, Kaohsiung, Taiwan

[4]Faculty of Renal Care, College of Medicine, Kaohsiung Medical University, Kaohsiung, Taiwan

[*]Corresponding author:chichi@cc.kmu.edu.tw

[+]These two contributed equally to this article.

Correspondence to: Chi-Chih Hung, MD. Division of Nephrology, Department of Internal Medicine, Kaohsiung Medical University Hospital, Kaohsiung Medical University, No.100 Tzyou First Road, Kaohsiung 807, Taiwan.

TEL: 886 7 3121101, FAX: 886 7 3122810

Support and Financial Disclosure Declaration

The authors declared no conflict of interests.

Supplement Table 1. Logistic regression for sodium <135 mEq/L in diuretic non-users

Variables	OR	95%CI	P
Age, year	0.997	0.990 to 1.005	0.497
Male	1.118	0.897 to 1.393	0.320
DM	1.044	0.812 to 1.343	0.736
CVD	1.310	1.030 to 1.667	0.028
Total body water			
1st quartile	1.466	0.934 to 2.301	0.096
2nd quartile	1.000	1.0	
3rd quartile	1.116	0.696 to 1.788	0.650
4th quartile	0.961	0.581 to 1.592	0.878
eGFR, ml/min/1.73m^2	0.992	0.987 to 0.998	0.008
Hb, g/dL	0.960	0.905 to 1.019	0.183
Albumin, mg/dL	0.433	0.355 to 0.529	0.000
CRPlog	1.123	1.014 to 1.243	0.026
HbA1c, %	1.217	1.143 to 1.295	0.000
UPCR log	0.993	0.810 to 1.219	0.950
Mean BP, mmHg	1.006	0.998 to 1.013	0.125
BMI, kg/m^2	0.939	0.914 to 0.966	0.000

Adjusted for age, gender, eGFR, diabetes mellitus, cardiovascular disease, mean blood pressure , HbA1c, hemoglobin, albumin, cholesterol, log-transformed urine protein to creatinine ratio, log-transformed C-reactive protein, body mass index, ACEI/ARB, anti-HTN agens, OAD agents and statins.

Supplement Table 2. Associations between serum sodium and outcomes in diuretic non-users

	Na (mEq/L)				P for interaction#
	**<135**	**135-138**	**138-141**	**>141**	
**Patients' Number**	651	1093	1430	583	
**Renal replacement Therapy**					
Event	236 (36.3%)	307 (28.1%)	290 (20.3%)	116 (19.9%)	
Unadjusted HR	1.35 (1.14-1.59)*	1 (reference)	0.63 (0.54-0.74)*	0.59 (0.48-0.73)*	
Adjusted HR	0.94 (0.79-1.13)	1 (reference)	0.94 (0.80-1.11)	1.01 (0.81-1.25)	0.037
**All-cause mortality**					
Event	157 (24.1%)	168 (15.4%)	149 (10.4%)	87 (14.9%)	
Unadjusted HR	1.55 (1.25-1.92)*	1 (reference)	0.63 (0.50-0.79)*	0.88 (0.68-1.14)	
Adjusted HR	0.93 (0.74-1.16)	1 (reference)	0.81 (0.65-1.01)	0.99 (0.76-1.29)	0.329
**Cardiovascular event**					
Event	112 (17.2%)	123 (11.3%)	131 (9.2%)	49 (8.4%)	
Unadjusted HR	1.66 (1.36-2.01)*	1 (reference)	0.72 (0.59-0.87)*	0.81 (0.64-1.03)	
Adjusted HR	0.98 (0.80-1.20)	1 (reference)	0.91 (0.75-1.11)	0.88 (0.69-1.12)	0.294

Adjusted for age, gender, eGFR, diabetes mellitus, cardiovascular disease, mean blood pressure , HbA1c, hemoglobin, albumin, cholesterol, log-transformed urine protein to creatinine ratio, log-transformed C-reactive protein, body mass index, ACEI/ARB, anti-HTN agents, OAD agents, statins, integrated CKD care and causes of renal diseases.

** ($p < 0.05$) indicates a significantly different from reference group.# interaction between diuretic use and serum sodium.*

Supplement table 3. Associations between serum sodium and outcomes in diuretic users.

	Na (mEq/L)		
	**<135**	**135-141**	**>141**
**Renal replacement therapy**			
Unadjusted HR	1.49 (1.21-1.85)*	1	1.15 (0.90-1.46)
Adjusted HR	1.35 (1.08-1.69)*	1	1.16 (0.96-1.41)
**All-cause mortality**			
Unadjusted HR	1.40 (1.03-1.90)*	1	1.45 (1.06-1.99)*
Adjusted HR	1.18 (0.86-1.63)	1	1.47 (1.07-2.02)*
**Cardiovascular event**			
Unadjusted HR	1.42 (1.11-1.81)*	1	1.21 (0.93-1.58)
Adjusted HR	1.10 (0.85-1.43)	1	1.19 (0.91-1.56)

Adjusted for age, gender, eGFR, diabetes mellitus, cardiovascular disease, mean blood pressure , HbA1c, hemoglobin, albumin, cholesterol, log-transformed urine protein to creatinine ratio, log-transformed C-reactive protein, body mass index, ACEI/ARB, anti-HTN agents, OAD agents, statins, integrated CKD care and causes of renal diseases.

** ($p < 0.05$) indicates a significantly different from reference group.# interaction between diuretic use and serum sodium.*

Figure Legends

Supplement figure 1. Association between serum sodium and renal replacement therapy by restricted cubic spline model in diuretic non-users.

Supplement figure 2. Association between serum sodium and all-cause mortality by restricted cubic spline model in diuretic non-users.

Supplement figure 3. Association between serum sodium and cardiovascular event by restricted cubic spline model in diuretic non-users.

Supplement figure 4. Association between serum sodium and renal replacement therapy by restricted cubic spline model with different knots in diuretic users.

Supplement figure 5. Association between serum sodium and all-cause mortality by restricted cubic spline model with different knots in diuretic users.

Supplement figure 6. Association between serum sodium and cardiovascular event by restricted cubic spline model with different knots in diuretic users.

Supplement figure 1.

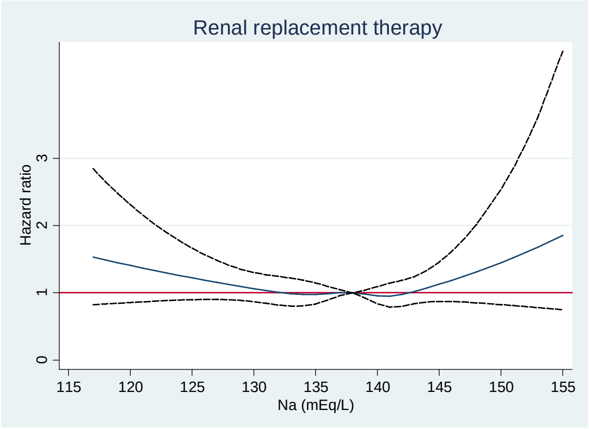

Supplement figure 2.

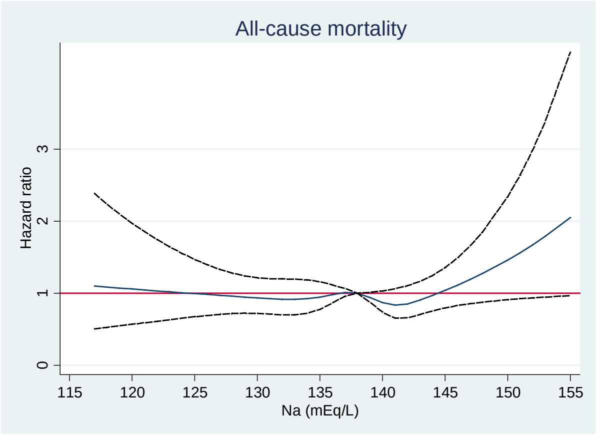

Supplement figure 3

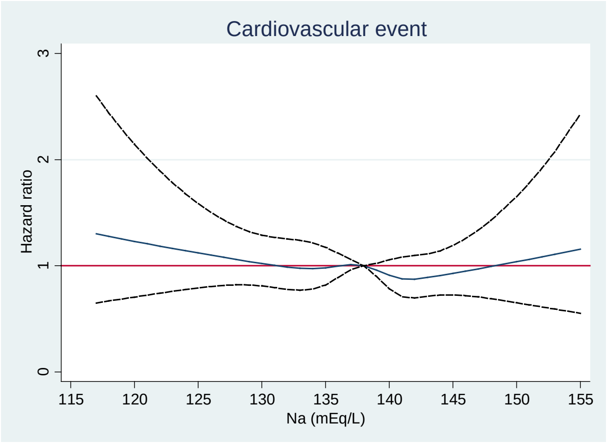

Supplement figure 4.

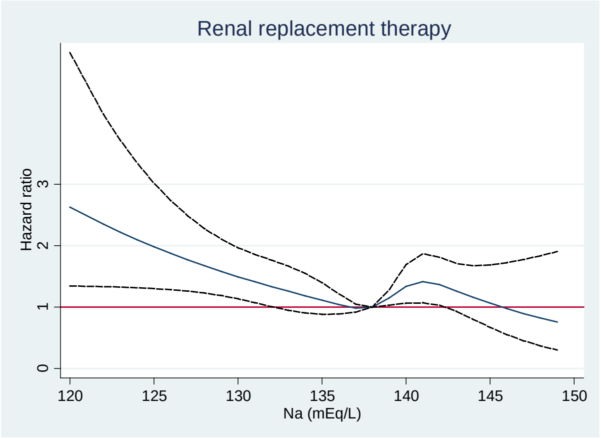

Supplement figure 5.

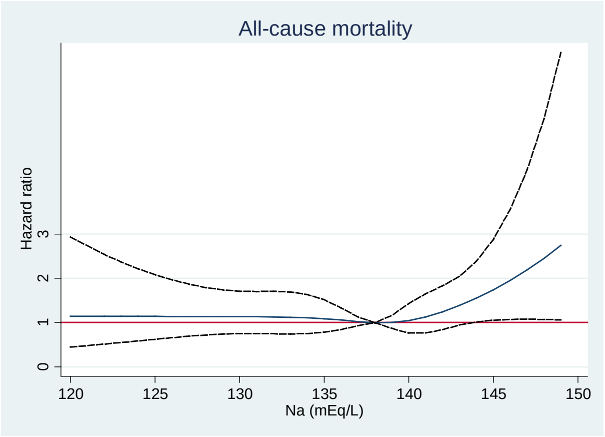

Supplement figure 6

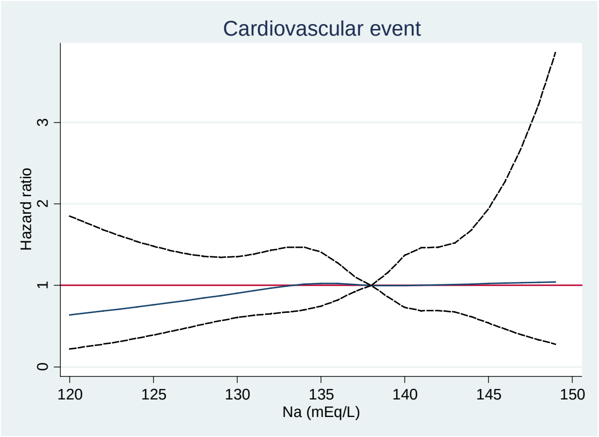

Supplement: Supplementary Information [file srep36817-s1.pdf]
